# *In Silico* Analysis of Potential Off-Target Effects of a Next-Generation dsRNA Acaricide for Varroa Mites (*Varroa destructor*) and Lack of Effect on a Bee-Associated Arthropod

**DOI:** 10.3390/insects16030317

**Published:** 2025-03-19

**Authors:** Mariana Bulgarella, Aiden Reason, James W. Baty, Rose A. McGruddy, Eric R. L. Gordon, Upendra K. Devisetty, Philip J. Lester

**Affiliations:** 1 School of Biological Sciences, Victoria University of Wellington, Wellington 6012, New Zealand; aiden.reason@vuw.ac.nz (A.R.); james.baty@vuw.ac.nz (J.W.B.); rosemcgruddy89@gmail.com (R.A.M.); phil.lester@vuw.ac.nz (P.J.L.); 2 GreenLight Biosciences, Research Triangle Park, 9 Laboratory Drive, Durham, NC 27709, USA; eric.gordon@greenlightbio.com (E.R.L.G.); ukdevisetty@greenlightbio.com (U.K.D.)

**Keywords:** biopesticides, double-stranded RNA, varroa control, RNA interference, wax moth

## Abstract

Double-stranded RNA (dsRNA) biopesticides offer the potential for highly targeted pest control with minimal off-target impacts. Varroa mites (*Varroa destructor*) are an important pest of honey bees (*Apis mellifera*) that are primarily managed by synthetic pesticides. A next-generation treatment using a varroa-active dsRNA, vadescana, has been developed to target calmodulin expression in varroa. We evaluated the potential exposure of non-target species to vadescana. First, we assessed potential gene silencing effects on 39 arthropods with known genomes via bioinformatics. Three mite species, monarch butterflies (*Danaus plexippus*), fruit flies (*Drosophila melanogaster*), and European earwigs (*Forficula auricularia*) showed theoretical potential for off-target effects. These *in silico* results could be used to help inform risk assessments. Second, we conducted vadescana feeding trials on the greater wax moth (*Galleria mellonella*), a common beehive associate. There were no significant differences in wax moth reproduction, survival, or adult F2 wing length between vadescana-fed and control groups. Male F2 body weight was slightly but significantly lower in wax moths exposed to the highest vadescana dose, with no such effect observed in female moths. Calmodulin gene expression was unaffected in wax moths. Our hazard assessment of vadescana’s lethal and sublethal effects on wax moths indicates minimal impact following continuous dietary exposure far greater than any exposure that might be expected in the field, in line with the bioinformatics findings. This biopesticide appears highly varroa-specific and likely has fewer non-target effects than many current varroa control methods.

## Simple Summary

We examined potential off-target effects of a novel double-stranded RNA (dsRNA) biopesticide to control varroa mites within honey beehives. The potential exposure of non-target species within beehives calls for comprehensive evaluation. We employed a two-fold approach. First, via bioinformatics analysis, we assessed potential gene silencing effects on arthropods associated with beehives and arthropods of conservation concern. Second, we conducted dsRNA feeding trials on a common beehive-associated species, the greater wax moth (*Galleria mellonella*), to corroborate that no hits in the bioinformatics analyses indeed meant no dsRNA activity *in vivo*. Our findings indicate minimal impact on wax moths following continuous dsRNA dietary exposure, in line with the bioinformatics findings of no perfect matching siRNA hits. Our results are indicative that this dsRNA biopesticide appears highly varroa-specific and likely has fewer non-target effects compared to many of the currently available varroa control methods based on synthetic chemicals.

## 1. Introduction

The beekeeping and pollination industries rely heavily on the European honey bee (*Apis mellifera*) for pollination and honey production services [1]. Currently, one of the greatest threats to honey bee health is the parasitic mite, *Varroa destructor* [2,3]. This mite (hereafter referred to as varroa) is particularly detrimental to honey bees because it vectors bee viruses [4,5], such as deformed wing virus, associated with overwinter mortality of honey bee colonies worldwide [5,6]. Varroa further engages in chemical mimicry, manipulates the bee host, swiftly disperses between and within colonies, and has evolved resistance to acaricides (reviewed by [7]), making the mite a difficult pest to control. Varroa is closely linked to its honey bee host and lacks a free-living stage [8]. The mite life cycle is split into two distinctive phases, the reproductive phase inside honey bee brood cells where a foundress mite raises her young, and the dispersal phase, where mature female mites disperse and feed on adult bees [7]. 

The most common control method for varroa is the use of chemical miticides such as the pyrethroids, flumethrin and tau-fluvalinate; the organophosphate coumaphos; and the formamidine amitraz; or alternative non-synthetic chemicals such as oxalic acid, thymol, and formic acid [8,9,10]. Resistance to synthetic acaricides has been reported frequently [11,12,13,14,15,16,17], rendering them ineffective in some areas, and both synthetic chemicals and non-synthetic phenols and organic acids can be detrimental to honey bees through sub-lethal and stress-related effects [18,19,20]. Therefore, novel modes of action to control varroa and varroosis in beehives are needed.

One alternative to broad-spectrum insecticides for pest control is the use of RNA interference or RNAi [21,22,23,24,25]. Double-stranded (dsRNA) biopesticides can be designed to enable selective targeting of insect pest species. dsRNA biopesticides work through the RNAi mechanism whereby specific messenger RNA (mRNA) transcripts are targeted by small interfering RNAs (siRNAs) and silenced via nuclease activity or translational repression. In insects, the siRNA pathway is activated when dsRNA molecules are recognized in the cytoplasm and processed into siRNAs of 21–25 nucleotides by the enzyme Dicer-2 [26]. Next, Argonaute proteins assemble with the siRNA to form an RNA-induced silencing complex (RISC) that targets the destruction of the endogenous mRNA complementary to its guide strand, resulting in specific knockdown of target proteins [23]. RNAi, when used to design dsRNA biopesticides, should target a gene within specific pests by identifying regions on the pest mRNA that have little or no sequence identity with mRNA of non-target species [27]. The availability of novel, RNA-based, and sustainable insect/mite management biopesticides would be advantageous due to high species-specificity and a relatively low environmental impact. A sprayable form of RNAi-based biopesticide is already commercially available against the Colorado potato beetle [24,28].

Garbian et al. [21] demonstrated that dsRNA has potential for varroa control. GreenLight Biosciences Inc. (Durham, NC, USA) has developed a dsRNA biopesticide to control varroa mites within beehive brood cells [29,30]. The treatment consists of a synthetic dsRNA identical to a region of the calmodulin gene in varroa mites. The new product is called Norroa^TM^ with the active ingredient given the common name vadescana (CAS# 2643947-26-4). Calmodulin is a Ca^2+^-binding protein expressed in eukaryotic cells where it signals pathways that regulate crucial processes such as growth, proliferation, and movement. In arthropods, calmodulin is also known to play a critical role in the uptake of vitellogenin during egg production [31]. It is a relatively small protein, only 149 amino acid residues in *V. destructor* and 148 residues in vertebrates [32,33]. Vadescana is provided to honey bee colonies in a pouch filled with a sucrose-based solution. One side of the pouch is perforated and placed on top of the brood frame from where the nursing bees transfer the fluid to their brood. Researchers have previously observed that the vadescana sequence shares a 21-base pair match with monarch butterfly, *Danaus plexippus*. However, feeding trials with varroa-active dsRNA targeting calmodulin found no off-target effects in these butterflies, with no mortality and no sublethal effects of this dsRNA treatment [27].

The objectives of our study were twofold: (1) to use bioinformatics to examine the potential for off-target effects of vadescana on a variety of arthropods known to be associated with beehives and on insect species of conservation concern; and (2) to experimentally determine if vadescana presented off-target effects in the greater wax moth, *Galleria mellonella*, by conducting feeding assays in the laboratory. The greater wax moth was selected for bioassay assessments due to its close association with managed honey bee colonies and it has been described as ubiquitously distributed wherever beekeeping occurs, feeding on honey, pollen, and honey bee brood [34]. Wax moth larvae have previously been reported to be susceptible to dsRNA produced by ingested bacteria [35,36]. We compared differences in calmodulin gene expression, survival, and morphology between wax moth fed either one of two concentrations of vadescana, a non-targeting dsRNA, or a control group consuming a no-dsRNA treatment consisting of a sugar solution.

## 2. Materials and Methods

### 2.1. Predicting Off-Target Effects Using Bioinformatics

Our first objective was to bioinformatically predict potentially active siRNAs of the synthetically produced 402 bp dsRNA targeting the calmodulin gene of varroa mites, vadescana (calmodulin GenBank accession number XM_022799184.1) [29]. We used the software si-Fi21 v.1.2.3.-0008 [37] to predict the potential for off-target effects of vadescana on genomes of non-target organisms. The off-target searching pipeline begins by splitting a dsRNA sequence into all possible x-mers (x = selected length of the siRNA), and then the algorithm compares the x-mers (in the forward and reverse orientations) to a database for matching sequences. Next, the siRNA efficiency of each hit is calculated using high sensitivity parameters. The off-target prediction uses the high-sensitivity mode settings for finding as many putative off-targets as possible while minimizing the false-positive signals [37]. Combined analysis of the siRNA sequence and target site allows for defining siRNA with putatively high efficiency (see Figure 1 in [37]). The algorithm first determines which strand is the guide or antisense by applying strand selection rules. The guide strand will be chosen by argonaute proteins and retained in RISC, making it fully functional [38,39]. Next, the algorithm calculates the probability of local accessibility on the target to predict whether the siRNAs are targeting accessible parts of the target RNA. The combination of both selection criteria increases the prediction power for RNAi selectivity [37]. Endonucleolytic cleavage of mRNA by the RISC complex requires pairing of the guide strand to the target, but not all regions of the target mRNA are equally accessible to siRNAs; for example, if the target site is covered by local secondary structures. Thus, not all hits will be efficient in initiating a silencing response [37]. The si-Fi21 software internally uses the program Bowtie [40] to search a database for matching sequences due to its speed and memory efficiency. Furthermore, we used an alternate program bwa-aln from the BWA package [41] to conduct an exhaustive search on both genomes and transcriptomes and to confirm the results from si-Fi21. Because we did not observe transcriptomic hits that were not present in genomes and because only a subset of species possessed known transcriptomes, efficacious siRNAs were evaluated by si-Fi21 on genome sequences only.

The relevant length for determining the specificity of any long dsRNA sequence depends on the length of the products processed from exogenous dsRNA by the Dicer enzyme(s) present in any particular species. In arthropods, small RNA sequencing studies of exogenous viral or other dsRNA sequences have shown variability in the size of these Dicer products across different organisms. In spider mites, the size of Dicer-processed exogenous dsRNA fragments was primarily 20–22 nt in length [42]. Summarizing across various groups of insects, representative moth species (Lepidoptera) possessed Dicer products of primarily 20 nt in length [43], various beetles (Coleoptera) of primarily 21 nt [43,44], while hemipterans, orthopterans, hymenopterans, and dipterans possessed Dicer products primarily of 22 nt or an even mix of 21–22 nt [43,45,46,47]. Based on the above and to simplify our analysis, we chose to use a length of 21 nt for determining theoretical off-target sequences in non-target organisms allowing no nucleotide mismatches.

We identified 76 arthropod species that were either commonly associated with beehives [48] or were of conservation concern, and also included other arthropods whose genomes are known (Table S1). The genomes and transcriptomes of 39 of the species on the list were available to download from the NCBI Genome Database as of 20 December 2023 (Table S1). Since siRNA efficacy ultimately depends on sequence homology at the mRNA level (e.g., distant exons that are later spliced together in mature mRNA), comparing 21 nt hits to the transcriptome, rather than the genome, is preferable. However, transcriptomes were not available for all non-target species. Thus, for the current off-target analysis, in addition to transcriptomes, we also evaluated available genomic resources of non-target organisms. For genomic resources, we counted occasions in which the same 21 nt sequence matched multiple locations in a genome, but for transcriptomes where the same genomic coding section may be present in multiple isoforms, we only counted unique 21 nt hits. For genome sequences, we compared the vadescana 402 bp sequence against each genome and calculated the total number of siRNA hits and how many of those hits could efficiently activate the siRNA machinery of the cell using si-Fi21 software. If a genome had an effective hit at size 21 nt, then we determined which genes would potentially be targeted using the published genomes, along with its location, such as exon, intron, or the exon–intron boundary junction. All genomic and transcriptomic resources were evaluated with BUSCO [49] using the orthologs from OrthoDB v9.1 [50] of the most closely related lineage (results in Table S2) to determine the estimated completeness of the resource.

### 2.2. Insect Husbandry and dsRNA Feeding Treatments

#### 2.2.1. dsRNA Setup/Dilution

All dsRNA solutions used in the feeding trials were synthesized and provided by GreenLight Biosciences, Inc. (Durham, NC, USA). We included four treatments in our bioassays: (1) 60% sucrose aqueous solution and 0 g/L of dsRNA (no dsRNA blank); (2) 2 g/L of a non-targeting dsRNA identical to a segment of the MPK4a gene in soybean (*Glycine max* mitogen-activated protein kinase MPK4a—GenBank accession number NM_001352963.1) selected as a negative control; (3) 2 g/L of vadescana in 60% sucrose solution; and (4) 4 g/L of vadescana in 60% sucrose solution. These concentrations were chosen because they represent those of the proposed end-use rate of the product.

#### 2.2.2. Wax Moths

**Figure 1:**
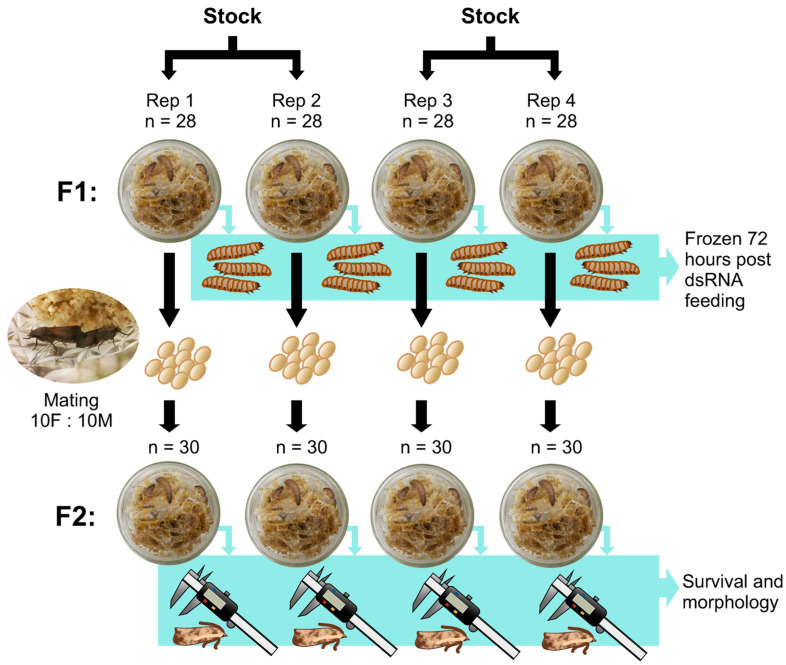
Diagram showing the experimental design for measuring survival, reproduction and morphology of wax moth larvae continually fed dsRNA-based diets over two generations. We conducted four replicates of each feeding trial. The F1 generation consisted of 28 larvae per replicate (~8–10 day-old), and 25 of these grew to adulthood, mated, and produced the F2 generation. Three larvae from each jar were frozen 72 h after dsRNA feeding started. For the F2 generation, 30 wax moth larvae were reared to adulthood per replicate, weighed, and wing length measured upon adult emergence. Illustrations by Aiden Reason, photographs by Mariana Bulgarella.

We sourced wax moth larvae from BioSuppliers Limited (Auckland, New Zealand). The rearing conditions were 27 ± 1 °C, 30 ± 10% relative humidity, and a light/dark photoperiod of 16:8 h. Upon arrival at the laboratory, ~8–10 day-old larvae [51] were randomly separated into four treatments in groups of 28 larvae each to make up the F1 generation (Figure 1). Replicates 1 and 2 originated from the same stock and replicates 3 and 4 originated from the same stock (different from replicates 1 and 2). We modified artificial diets previously used in wax moth research [52,53]. In a sterilized glass beaker, we thoroughly mixed the following ingredients: 15 g of glycerin (Pure Nature, Auckland, New Zealand), 15 g of beech forest honey (Manuka Doctor, Auckland, New Zealand), 30 g of dsRNA of the corresponding treatment described below, 5 mL of water, 50 g of Farex© original multigrain cereal (fine grains, 6+ months, H.J. Heinz Company, Victoria, Australia) and 4 g of dried yeast (Tasti, Auckland, New Zealand). This recipe was enough to supply food to the individual glass jars and excess food was disposed. As above, the treatment groups varied in the amount of dsRNA included in the diet: (1) 0 g/L of dsRNA (no dsRNA control), (2) 2 g/L of non-targeting dsRNA, (3) 2 g/L of vadescana, and (4) 4 g/L of vadescana. We ran four replicates of each trial, and three individual larvae from each replicate (jar) were frozen 72 h after feeding started for calmodulin expression analysis (see below). 

The sample size for the survival data was 25 larvae per treatment group for the F1 generation (Figure 1). The first-generation larvae were kept in 237 mL wide mouth glass jars with mesh in the metal lids (Ball, Westminster, CO, USA). We repeated the dsRNA feeding for second generation larvae that were kept in 500 mL wide mouth glass jars with mesh in the lids (Agee, Sydney, Australia) in groups of 30 larvae each. Larvae food was replenished twice a week until pupation. Once F1 individuals pupated to adulthood, we moved the adults to new glass jars in groups of 20 individuals (1:1 sex ratio), where females laid eggs and the eggs developed to adulthood. The excess F1 adults that did not contribute to the next generation and all F2 adults were frozen the day they emerged. All adult wax moths from F2 were weighed to the nearest 0.001 g using a precision scale (Sartorius Entris, Göttingen, Germany) and the length of the left wing was measured to the nearest mm with digital calipers (Fullers, Pointe-Claire, QC, Canada). This experimental design reflects a worst-case scenario in which consecutive wax moth generations (F1 larvae: ~1 month and F2 larvae: ~1 month) were continuously exposed to vadescana in high concentrations—exposure vastly exceeding any exposure that could reasonably be expected to occur in the field.

To estimate the survival of wax moths into adulthood following dsRNA feeding, we computed the proportion of wax moths that pupated to adulthood relative to the proportion of wax moths that entered pupation (not to the original number of larvae in each jar) using binomial generalized linear models as the data were not normally distributed. We were unable to use the original number of larvae per jar because many of the tiny larvae escaped the jars through the mesh lids. To compare the F2 adult wax moth body weight and wing length, separately, for individuals from each sex, treatment, and replicate, we used a linear mixed model with sex and treatment as factors, with replicate as a random effect nested within treatment with the package ‘nlme’ [54]. Whenever there were no significant interactions (*p* > 0.05) in the model, we removed them and ran a simplified model.

#### 2.2.3. RNA Extraction, Reverse Transcription, and Quantitative PCR

For the bioassays, we collected at random three ~11–13-day-old wax moth larvae from the F1 generation per treatment group (*n* = 4) and per replicate (*n* = 4) 72 h after dsRNA feeding started. We chose to freeze the larvae at 72 h post dsRNA feeding based on previous insect research that reported silencing effects of orally delivered dsRNA 72 h after feeding started [55,56]. The three individual larvae from the same treatment group were frozen in a tube at –80 °C and RNA was extracted from these three individuals pooled together. The total sample size was sixteen samples (one sample per treatment for each of the four replicates).

To determine gene expression changes, we extracted RNA from wax moths using a C-TAB/chloroform-style extraction protocol as follows. Each sample was homogenized in a microcentrifuge tube containing 1 mL of GENEzol plant DNA reagent (Geneaid Biotech, New Taipei City, Taiwan), 5 µL of β-mercaptoethanol (Sigma Aldrich, Burlington, MA, USA), and two 5 mm stainless steel beads in a Precellys Evolution homogenizer (Bertin Instruments, Montigny-le-Bretonneux, France). Next, RNA was isolated using a 24:1 chloroform–isoamyl alcohol mixture (BioUltra, Sigma–Aldrich, Burlington, MA, USA) followed by isopropanol precipitation (BioReagent, Sigma–Aldrich, Burlington, MA, USA), and 70% ethanol purification (VWR Chemicals, Leicestershire, UK). RNA was eluted in 100 µL of nuclease-free water (Ambion, Austin, TX, USA). The RNA was quantified using a NanoPhotometer NP80 (Implen, Bayern, Germany), and treated with DNase I (Zymo Research, Irvine, CA, USA) to remove gDNA. Then, 250 ng of RNA was reversed transcribed with Quanta qScript cDNA SuperMix (Quantabio, Beverly, MA, USA) in 10 µL reactions following the manufacturer’s instructions.

Calmodulin gene expression levels in dsRNA-fed wax moths versus control individuals was measured by quantitative polymerase chain reaction (qPCR). We designed primers for the calmodulin gene for wax moths (CaM-F-wm AAAGAGTTGGGCACCGTGAT and CaM-R-wm GGGAAGTCTATCGTGCCGTT). These primers are not complementary to the vadescana sequence so they cannot amplify residual reverse-transcribed dsRNA fed to the insects. Sample cDNA was diluted to 2 ng/μL with nuclease-free water and 8 μL were combined with a 12 μL mix containing PowerUp SYBR Green Master Mix (Applied Biosystems/Thermo Fisher Scientific, Waltham, MA, USA) and the forward and reverse primers (final concentrations of 300 nM). Quantitative PCR was conducted in 96-well plates on a QuantStudio 7 Flex Real-Time PCR platform (Applied Biosystems/Thermo Fisher Scientific, Waltham, MA, USA). Fast cycling conditions consisted of 50 °C for 2 min; 95 °C for 2 min; 40 cycles of 95 °C for 1 s and 60 °C for 30 s. Fluorescence was measured at the 60 °C step and quantification cycle (Cq) values averaged from two technical replicates were used to normalize gene expression data relative to two internal reference genes, elongation factor 1-α (EF1) and 18S rRNA [35,57], chosen based on their stability in previous research.

Relative gene expression was calculated by normalizing the values of raw calmodulin mRNA expression to the average of the reference gene mRNA expression values using the equation (2ˆ(−Cq calmodulin))/average of (2ˆ(−Cq EF1)) and (2ˆ(−Cq 18S rRNA)) [58,59,60]. Relative gene expression was calculated using the average of the experimental samples divided by the average of the control samples (no dsRNA control) and is reported along with the standard error of the mean. Due to the non-normality of the qPCR data, we compared the differences in gene expression with non-parametric Kruskal–Wallis ANOVA tests [61] followed by Dunn post hoc tests where appropriate with *p* values adjusted for multiple comparisons with the Benjamini–Hochberg method.

## 3. Results

### 3.1. Predicting Off-Target Effects Using Bioinformatics

**Figure 2:**
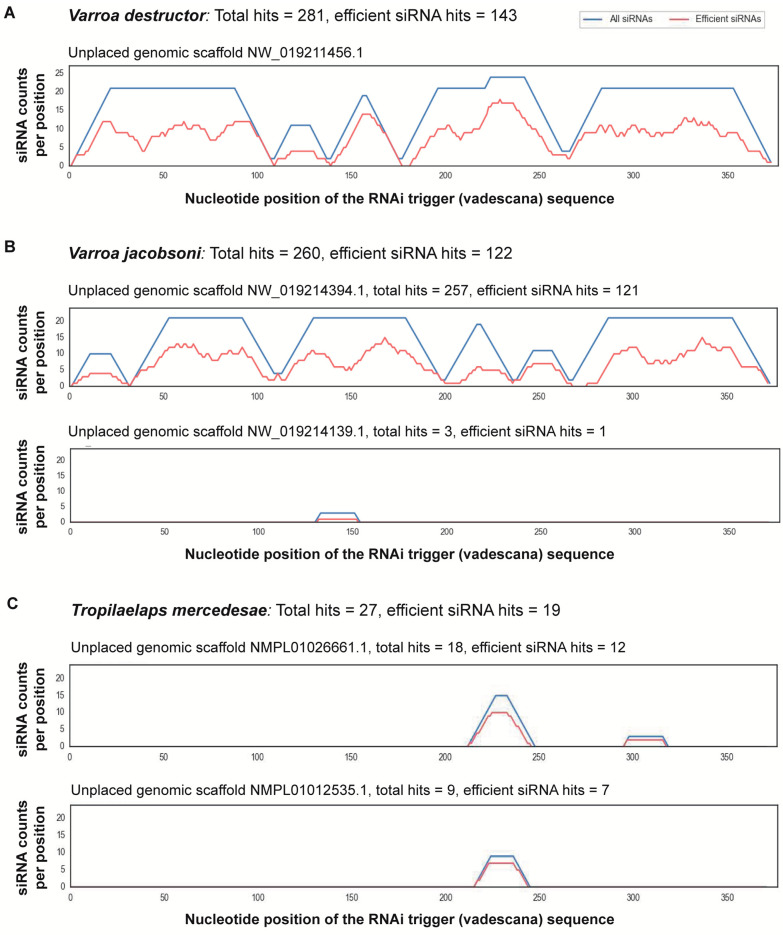
Results from si-Fi21 [37] off-target analysis of the vadescana sequence on three mite species when the siRNA size is set to 21 nt. Combined analysis of the siRNA sequence and target site allows predicting siRNA with high efficiency. The algorithm first determines which is the guide strand by applying strand selection rules. Next, it calculates the probability of local accessibility on the target to predict whether the siRNAs are targeting accessible parts of the target RNA [37]. (**A**) The total number of hits for *V. destructor* was 281, with 143 of these hits thought to be efficient, all within an unplaced genomic scaffold, GenBank accession NW_019211456.1, which contains the calmodulin-like mRNA (XM_022799184.1). (**B**) *Varroa jacobsoni* showed a total of 260 hits, with 122 predicted as efficient. The hits were on two different genomic regions; 257 hits fell in an unplaced genomic scaffold in the *V. jacobsoni* genome (GenBank accession NW_019214394.1), which contains the calmodulin-like gene (XM_022850852). Three additional hits were on another unplaced genomic scaffold, NW_019214394.1, in the *V. jacobsoni* genome, with only one of these predicted to be an efficient siRNA hit. (**C**) For the mite *Tropilaelaps mercedesae*, a total of 27 hits were found, with 19 of those thought to be efficient siRNA hits, located on two different unplaced genomic regions; 18 hits were in the NMPL01026661.1 genomic scaffold in the *T. mercedesae* genome and 12 of these are predicted to be efficient, while 9 hits were predicted on a second unplaced genomic scaffold (GenBank accession NMPL01012535.1), with seven of these supposed to be efficient at initiating a RNAi response.

When analyzing siRNA of 21 nt length, as expected, we found a high number of potential siRNA hits for the two varroa mite species: 281 and 260 for *V. destructor* and *V. jacobsoni*, respectively (Table 1). About half of those hits were predicted as being efficient in initiating a siRNA silencing response within the cell, and all matches fell within coding exons. The 281 hits for *V. destructor* were within an unplaced genomic scaffold (GenBank accession NW_019211456.1) on the *V. destructor* genome that contains the calmodulin-like mRNA sequence (XM_022799184.1; Figure 2). For *V. jacobsoni*, 257 hits were within an unplaced genomic scaffold in the *V. jacobsoni* genome (GenBank accession NW_019214394.1), which contains the calmodulin-like mRNA sequence (XM_022850852). Three additional hits for *V. jacobsoni* were for NW_019214394.1, a different unplaced genomic scaffold in the *V. jacobsoni* genome (Figure 2).

For the remaining 37 non-target genomes of arthropods that were available for analysis, 31 species showed no potential siRNA hits. There were six species that contained predicted siRNA hits at 21 nt length: the monarch butterfly *Danaus plexippus*, the fruit fly *Drosophila melanogaster*, the European earwig *Forficula auricularia*, and three species of mites (*Stratiolaelaps scimitus*, *Tetranychus urticae*, and *Tropilaelaps mercedesae*) (Table 1). The number of predicted siRNA hits of the mite species was not surprising given their relatedness to varroa. For *T. mercedesae* there were a total of 27 hits on two different unplaced genomic regions, with 19 of those thought to be efficient siRNA hits; 18 hits were in genomic scaffold NMPL01026661.1 in the *T. mercedesae* genome and 12 of these were predicted to be efficient. There were nine hits on a second unplaced genomic scaffold (GenBank accession NMPL01012535.1), with seven of these supposed to be efficient in initiating an RNAi response. In the case of the monarch butterfly, a single siRNA hit was predicted to have the potential to effectively silence the butterfly’s calmodulin gene, a result which has previously been examined by Krishnan et al. [27]. These authors found no evidence of any measurable effect of the dsRNA on life-history traits of monarch butterflies even at ten-fold higher doses than proposed to be used in the treatment of honey bee hives [27]. For the fruit fly, we found that five siRNAs were predicted to have the potential to be efficient in silencing different transcript variants of the calmodulin gene. Similarly, one hit for the European earwig was found to have the potential of silencing an annotated gene, which appears to encode a calmodulin protein when compared to other sequences via BLAST v.2.11.0+.

All transcriptomes and the majority of genomes were found to have greater than 90% estimated completeness based on BUSCO scores. Only one resource was found to be substantially incomplete, the genome of the house cricket, *Acheta domestica*, with an estimated completeness of only 48.7%. However, we confirmed that the house cricket assembly did possess a calmodulin ortholog (coding sequence present on scaffold JAAVVF010534026.1 at coordinates 11023–11672 with one intron present) and that it shared no 21 nt segments in common with the vadescana sequence. While it is possible that other genes absent in this assembly or others could contain 21 nt segments matching the dsRNA sequence, all 21 nt hits found in other organisms originated from the coding sequence of a calmodulin homolog only (confirmed by characterizing unannotated regions in assemblies with hits via BLAST).

Considering the results from the bioinformatics analyses and the likelihood of arthropods to be exposed to vadescana within bee hives, we further investigated one species that has been found to be commonly and widely associated with beehives: the greater wax moth [34], which does not possess any 21 nt hits. Experiments were conducted to determine if orally ingested dsRNA had any effect on its survival and growth or was efficient at silencing the calmodulin gene of this species.

### 3.2. dsRNA Feeding Treatments

#### Wax Moth Survival, Morphology, and Calmodulin Expression

When comparing survival to adulthood for the F1 generation, we found no significant differences in the proportion of wax moth individuals that survived (glm, *p* > 0.05 for all three comparisons). The proportion of F1 wax moths that pupated to adulthood was 94.0% for the no dsRNA treatment (*n* = 84), 89.5% for the non-targeting dsRNA treatment (*n* = 86), 96.4% for the 2 g/L of vadescana treatment (*n* = 84) and 93.9% for the 4 g/L of vadescana treatment (*n* = 82; Table 2). We also did not observe any obvious differences in wax moth reproduction among the four treatments. Even though we did not quantify the number of eggs laid, all jars had high numbers of eggs that turned into viable larvae with no dead larvae observed (although wax moths are cannibalistic, so no dead individuals were expected to remain in the jars).

There were also no significant differences between treatments in the proportion of F2 wax moths that pupated into adulthood (glm, *p* > 0.05 for all three comparisons). The proportion of wax moths that pupated to adulthood in the F2 was 97.5% for the control treatment (*n* = 119), 95.4% for the non-targeting dsRNA treatment (*n* = 109), 100% for the 2 g/L of vadescana treatment (*n* = 120), and 99.1% for the 4 g/L of vadescana treatment (*n* = 111; Table 2).

**Figure 3:**
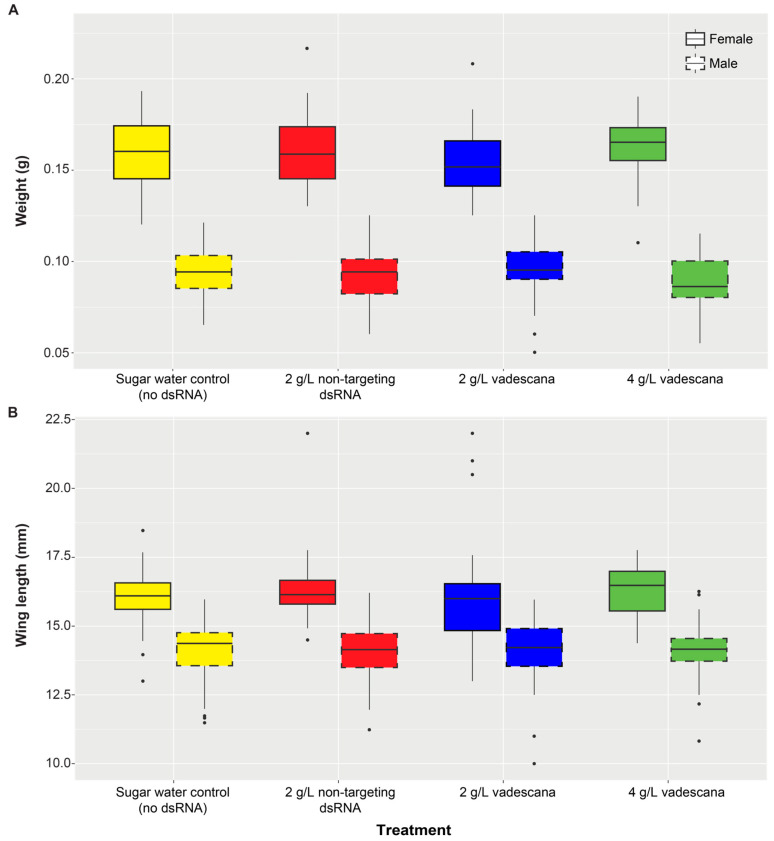
Morphological measurements for female and male F2 adult wax moths, measured the day after adult emergence, following a continuous dsRNA-based diet during larval development. The different colors correspond to the four different treatments. (**A**) Body weight. We found a significant effect of treatment for the 4 g/L of vadescana when compared to the control group (*t* = 1.98, df = 426, *p* = 0.048); sex had a significant effect (*p* < 0.001); and an interaction between males and the 4 g/L of vadescana treatment (*t* = −2.73, df = 426, *p* = 0.006). (**B**) Wing length. Treatment had no statistically significant effect, with sex having a significant effect (*p* < 0.001). Each box shows the lower and upper quartiles, the thick black line within the box is the median, the error bars are the minimum and maximum values, respectively, and the dots represent outlier values.

For wax moth adult body weight, there was a significant effect of treatment, where the 4 g/L of vadescana treatment group differed significantly from the control group (*t* = 1.98, df = 426, *p* = 0.048), a significant effect of sex (*p* < 0.001) with an interaction between males and 4 g/L of vadescana treatment (*t* = −2.73, df = 426, *p* = 0.006; Figure 3A). The male wax moths fed 4 g/L of vadescana weighed slightly less (0.088 ± 0.001 g, *n* = 55) than the control males (0.093 ± 0.002 g, *n* = 53). For adult wax moth wing length, there was no effect of treatment (glm, *p* > 0.05 for all comparisons) and a significant effect of sex (*p* < 0.001; Figure 3B). Sex differences in weight and wing length between male and female wax moths are well known with females being larger than males [53].

**Figure 4:**
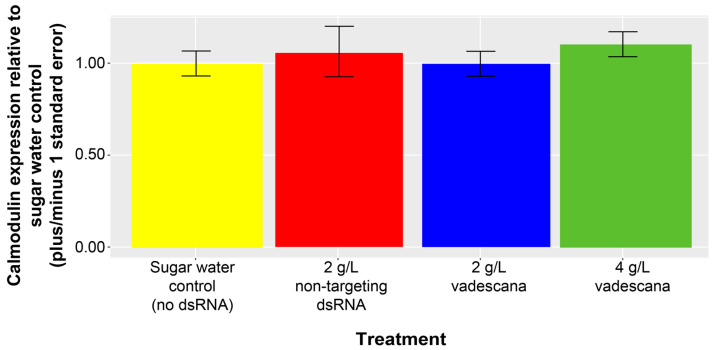
Relative calmodulin gene expression for wax moth fed dsRNA-based diets for three days. Each bar represents a treatment group and consists of four samples each (one sample is made up of three individual larvae from the same treatment jar pooled, *n* = 48 larvae, qPCR *n* = 16).

Relative calmodulin gene expression following vadescana consumption by F1 wax moth larvae was not significantly different than the no dsRNA control larvae for any treatment (Kruskal–Wallis χ^2^ = 3, df = 3, *p* = 0.391, Figure 4).

## 4. Discussion

In this study, we first determined the potential off-target effects of vadescana on the genome of 39 species of arthropods via bioinformatics. We implemented an approach based on sequence complementarity of the genome of non-target species to the vadescana sequence, comparing all possible 21 nucleotide stretches for a match. As expected, bioinformatics analyses comparing the 21 nt siRNA found many hits on the two conspecific varroa species, *V. destructor*, and *V. jacobsoni*, as the dsRNA query sequence is designed to target this pest genus. We also found some number of 21 nt hits for three other mite species, *Stratiolaelaps scimitus*, *Tetranychus urticae*, and *Tropilaelaps mercedesae*. Due to their relatedness to varroa, these mites have similar calmodulin gene sequences. In the case of *T. mercedesae*, 27 hits were found, with 19 of those thought to be efficient siRNA hits.

Any actual potential effects of vadescana on these mites or other arthropod species would require *in vivo* assays to confirm that vadescana could activate the RNAi pathway. *Stratiolaelaps scimitus* and *T. urticae* are not found in or near beehives and, as such, are unlikely to be exposed to vadescana. It is worth noting that the activity of vadescana toward many mite species within beehives could be beneficial. For example, tracheal mites (*Acarapis woodi*) are near-globally distributed pests that have been associated with substantial colony losses [62], and *Tropilaelaps* mites, such as *T. mercedesae*, are emerging pests of major concern, including as parasites of honey bees [63]. While it is unlikely that vadescana will have activity against these mites due to no (*A. woodi*) or a limited (*T. mercedesae*) number of 21 nt matches, this outcome was not tested in this work. Additionally, *V. jacobsoni* has become a significant pest in the Pacific region, with particularly devastating effects in *Apis mellifera* populations in Papua New Guinea and Fiji where management options remain limited [64]. Vadescana may be useful for the management of *V. jacobsoni*, and this avenue will require testing and further analysis.

The bioinformatic analyses determined that vadescana shares five 21 nt matches with the fruit fly, corresponding to five different transcript variants of the calmodulin gene (GenBank accessions NM_001259348.3, NM_001299408.1, NM_165870.2, NM_001259347.2, and NM_078986.3). It is worth noting that naked dsRNA was ineffective at activating a RNAi response when ingested by four *Drosophila* species [65]. The European earwig has one 21 nt match within a gene that is not annotated in the database (GenBank accession JALMQSO10000099.1) but represents a calmodulin ortholog in this species as revealed by comparison of the sequence to other known calmodulin genes. Controlled laboratory studies could investigate these findings in fruit flies and earwigs. However, neither of these species are found near or inside bee hives, and thus are unlikely to be exposed to vadescana. We only included these species in our bioinformatics analyses because their genomes were known, and the number of arthropod genomes available was very limited.

Finally, vadescana shares a 21 nt match with the calmodulin mRNA of monarch butterflies. When monarch butterflies were fed either a dsRNA molecule that targets the same region of *V. destructor* calmodulin as vadescana or a sequence with 100% sequence identity to the monarch vATPase A mRNA sequence, no mortality and no sublethal effects were found [27]. Krishnan et al. [27] concluded that monarch mRNA may be refractory to silencing by dsRNA or the dsRNA might be degraded by dsRNases, reducing its ability to silence mRNA. Lepidopterans are known to be refractory to RNAi, which implies off-target effects would be unexpected [27,66]. Furthermore, monarch butterflies would not be expected to enter a honey bee colony and be directly exposed to vadescana.

Analyses based only on sequence homology are likely to overestimate the likelihood of off-target effects, as each siRNA generated from a dsRNA will have low abundance [67]. There are many reasons why a non-target species with a transcript homologous to the dsRNA might be unaffected by it. Different species differ in tissue RNAi sensitivity due to differences in the core RNAi machinery composition, amplification of RNAi signals *in vivo*, and activation of dsRNA-degrading nucleases; the dsRNA might not be taken up or it might not be transported to the cell where the off-target transcript is expressed or when species are shown to be recalcitrant to RNAi. All of these processes would result in no quantifiable physiological effect of dsRNA consumption [66,67]. Thus, while bioinformatics can rule out the possibility of RNAi against a non-target organism based on sequence homology, the presence of 21 nt matches alone is not sufficient to confirm RNAi gene silencing in a species.

In addition to our bioinformatics analysis, we evaluated potential off-target effects of vadescana on the greater wax moth in controlled laboratory studies. While the greater wax moth is a known honey bee associate [68,69], our trials represented an extreme scenario in which the insects were exposed to high concentrations of the dsRNA throughout their lives. We found no significant effects of vadescana consumption when compared to moths fed a sugar solution on survival, reproduction, calmodulin expression, or wing length in four trial replicates involving two generations of wax moths. A significant effect of vadescana at 4 g/L was found for the weight of male wax moth adults, but not females. Males reared on the 4 g/L vadescana diet weighed slightly less than the control males fed the no dsRNA solution. This significant effect between treatments showing a biologically small effect size largely appeared driven by high sample numbers and low levels of variation. Overall, our results show that it would seem unlikely that greater wax moths would suffer adverse effects if they were exposed to vadescana through their diets in a hive environment.

Honey bees would clearly be exposed to vadescana or a similar dsRNA treatment if it were to be used for varroa control within a hive. Separate experiments have indicated no observed detrimental effects of vadescana on honey bee larvae and pupae [70]. In addition, Radio Frequency Identifier (RFID) tags were used to examine foraging behavior and the lifespan of adult bees when treated with this dsRNA [71]. This RFID study indicated vadescana treated adult bees exhibited significantly more foraging and slightly shorter duration trips than bees without varroa treatment or by bees from colonies treated with Apivar^®^ (an amitraz-based treatment). As an apparent consequence of this foraging behavior, vadescana-treated bees had an average lifespan of 25.4 days compared to amitraz treated hives with 28.7 days. Bees without varroa treatment lived for an average of 21.8 days [71]. These results indicate beneficial effects of vadescana for bees with no observed negative impact in comparison to colonies without mite control. These results with honey bees, in combination with the lack of predicted non-target effects on other organisms or wax moths as shown in this study, appear to confirm a high degree of specificity and potential.

## Figures and Tables

**Table 1:** Off-target prediction results for 39 arthropod species commonly associated with beehives. Our query was a synthetic dsRNA targeting the calmodulin gene of varroa mites (vadescana). For genomes, we counted occasions in which the same 21-mer sequence matched to multiple locations, but for transcriptomes where the same genomic coding section may be present in multiple isoforms, we only counted unique 21-mer matches. For genome resources, the si-Fi21 software [37] calculated the total number of siRNA hits and how many of those hits were predicted to be efficient at initiating a RNAi response within the cell for genome sequences. We used a 21 nt siRNA with no mismatches allowed. Results for varroa mites are shown in bold font. Note: species included are limited to those whose genomes were available on the NCBI Genome Database as of 20 December 2023. For hits to unannotated features, we characterized the surrounding genomic locus, finding that on all occasions the hit/s were present in a coding region of a calmodulin gene or different transcript variants of calmodulin.

Species	Common Name	GenBank or RefSeqGenome/ Transcriptome ID	21-Mer Hits	
			Genome (Total/ Efficient)	Transcriptome (Unique)
**Arachnida**				
Mesostigmata				
*Stratiolaelaps scimitus*	Stratiolaelaps mite	GCA_019614645.1	9/6	-
*Tropilaelaps mercedesae*	Tropilaelaps mite	GCA_002081605.1	27/19	18
***Varroa destructor***	Honey bee mite	GCF_002443255.1	**281/143**	**278**
***Varroa jacobsoni***	Varroa mite	GCF_002532875.1/GCF_002532875.2	**260/122**	**257**
Trombidiformes				
*Acarapis woodi*	Honey bee tracheal mite	GCA_023170135.1	0	-
*Tetranychus urticae*	Two-spotted spider mite	GCF_000239435.1	1/1	1
**Insecta**				
Blattodea				
*Blattella germanica*	German cockroach	GCA_000762945.2	0	-
Coleoptera				
*Aethina tumida*	Small hive beetle	GCF_024364675.1	0	0
Dermaptera				
*Forficula auricularia*	European earwig	GCA_024734495.1	1/1	-
Diptera				
*Drosophila melanogaster*	Fruit fly	GCF_000001215.4	10/5	10
*Hermetia illucens*	Black soldier fly	GCF_905115235.1	0	0
Hymenoptera				
*Apis cerana*	Asiatic honey bee	GCF_001442555.1	0	0
*Apis dorsata*	Giant honey bee	GCF_000469605.1	0	0
*Apis florea*	Little honey bee	GCF_000184785.3	0	0
*Apis mellifera*	Honey bee	GCF_003254395.2	0	0
*Bombus ignitus*	Fiery-tailed bumble bee	GCA_014825875.1	0	-
*Bombus impatiens*	Common eastern bumble bee	GCF_000188095.3	0	0
*Bombus lapidarius*	Red tailed bumble bee	GCA_936014575.1	0	-
*Bombus pascuorum*	Common carder bee	GCF_905332965.1	0	0
*Bombus sylvicola*	Forest bumble bee	GCA_019677175.1	0	-
*Bombus terrestris*	Buff-tailed bumble bee	GCF_910591885.1	0	0
*Linepithema humile*	Argentine ant	GCF_000217595.1	0	0
*Monomorium pharaonis*	Pharaoh ant	GCF_013373865.1	0	0
*Polistes canadensis*	Red paper wasp	GCF_001313835.1	0	0
*Polistes dominula*	European paper wasp	GCF_001465965.1	0	0
*Polistes fuscatus*	Common paper wasp	GCF_010416935.1	0	0
*Solenopsis invicta*	Red fire ant	GCF_016802725.1	0	0
*Vespa velutina*	Yellow-legged hornet	GCF_912470025.1	0	0
*Vespula germanica*	German wasp	GCA_905340365.1	0	-
*Vespula vulgaris*	Common wasp	GCA_905475345.1	0	0
Lepidoptera				
*Achroia grisella*	Lesser wax moth	GCF_030625045.1	0	0
*Danaus plexippus*	Monarch butterfly	GCF_009731565.1	1/1	1
*Galleria mellonella*	Greater wax moth	GCF_026898425.1	0	0
*Pieris rapae*	Cabbage white butterfly	GCF_905147795.1	0	0
*Vanessa cardiu*	Painted lady	GCF_905220365.1	0	0
Orthoptera				
*Acheta domestica*	House cricket	GCA_014858955.1	0	-
*Locusta migratoria*	Migratory locust	GCA_026315105.1	0	-
*Teleogryllus occipitalis*	Asian cricket	GCA_011170035.1	0	0
Phasmatodea				
*Clitarchus hookeri*	Smooth stick insect	GCA_002778355.1	0	-

**Table 2:** Survival of wax moth larvae fed on a dsRNA-based diet in the laboratory. Please note that the number of larvae that pupated does not necessarily reflect that the larvae/pupae died, as there were many instances of escaping larvae. To estimate survival, we compared the number of larvae that entered pupation to the number of pupated adults.

Treatment	Replicate	Starting Number of F1 Larvae/Number of Larvae That Pupated	Number of F1 Adults	Starting Number of F2 Larvae/Number of Larvae That Pupated	Number of F2 Adults
Control, no dsRNA	1	25/17	17	30/30	29
	2	25/17	16	30/29	27
	3	25/25	25	30/30	30
	4	25/25	21	30/30	30
2 g/L non-targeting	1	25/19	17	30/26	24
dsRNA	2	25/21	19	30/23	22
	3	25/25	24	30/30	30
	4	25/21	17	30/30	28
2 g/L of vadescana	1	25/19	17	30/30	30
	2	25/17	17	30/30	30
	3	25/24	23	30/30	30
	4	25/24	24	30/30	30
4 g/L of vadescana	1	25/19	17	30/22	22
	2	25/21	20	30/29	28
	3	25/22	21	30/30	30
	4	25/20	19	30/30	30

## Data Availability

The data that support the findings of this study are available from the corresponding author upon request.

## Supplementary Materials

The following supporting information can be downloaded at: https://www.mdpi.com/article/10.3390/insects16030317/s1, Table S1: Seventy-six species of arthropods commonly associated with beehives, of conservation concern, or whose genomes are known and were identified of interest for the determination of off-target effects of vadescana. The genomes of 39 of the species on the list were available to download from the NCBI Genome Database as of 20 December 2023 and were included in the bioinformatics analyses; Table S2: BUSCO evaluations of genomic and transcriptomic resources used for the determination of off-target effects of vadescana. C: Complete, S: Single copy, D: Duplicated, F: Fragmented, M: Missing, n: Number of genes in OrthoDb lineage.
