# Supplementary material for: In Silico Analysis of Potential Off-Target Effects of a Next-Generation dsRNA Acaricide for Varroa Mites (Varroa destructor) and Lack of Effect on a Bee-Associated Arthropod
Source: Insects. 2025 Mar 19;16(3):317. doi: 10.3390/insects16030317 (PMC11942661; doi:10.3390/insects16030317)
Supplement: Supplementary file 1 [file insects-16-00317-s001.zip › insects-3514171-supplementary.pdf]

Supplemental Material for In-silico analysis of potential off-target effects of a next-generation dsRNA acaricide for varroa mite (*Varroa destructor*) and lack of effect on a bee-associated arthropod

Mariana Bulgarella, Aiden Reason, James W. Baty, Rose A. McGruddy, Eric R. L. Gordon, Upendra K. Devisetty and Philip J. Lester

Supplemental Table S1. Seventy-six species of arthropods commonly associated with beehives, of conservation concern, or whose genomes are known were identified of interest for the determination of off-target effects of vadescana. The genomes of 39 of the species on the list were available to download from the NCBI Genome Database as of 20 December 2023, and were included in the bioinformatics analyses.

#	Species name	Common name	Rationale for inclusion	Genome available	Transcriptome available
	**Arachnida**				
	Araneae				
1	*Diaea spp.*	Flower spiders	Other	no	no
2	*Helpis minitabunda*	Jumping spider	Other	no	no
3	*Lycosa spp.*	Wolf spiders	Other	no	no
4	*Steatoda spp.*	Spiders	Other	no	no
	Mesostigmata				
5	*Stratiolaelaps scimitus*	Stratiolaelaps mite	Bee associate	yes	no
6	*Tropilaelaps clareae*	Tropilaelaps mite	Bee associate	no	no
7	*Tropilaelaps koenigerum*	Tropilaelaps mite	Bee associate	no	no
8	*Tropilaelaps mercedesae*	Tropilaelaps mite	Bee associate	yes	yes
9	*Tropilaelaps thaii*	Tropilaelaps mite	Bee associate	no	no
10	*Varroa destructor*	Honey bee mite	Bee associate	yes	yes
11	*Varroa jacobsoni*	Varroa mite	Bee associate	yes	yes
	Trombidiformes				
12	*Acarapis extrenus*	*Acarapis* mite	Bee associate	no	no
13	*Acarapis dorsalis*	*Acarapis* mite	Bee associate	no	no
14	*Acarapis woodi*	Honey bee tracheal mite	Bee associate	yes	no
15	*Tetranychus urticae*	Two-spotted spider mite	Bee associate	yes	yes
	**Insecta**				
	Blattodea				
16	*Blattella germanica*	German cockroach	Other	yes	

17	*Celatoblatta spp.*	Cockroaches	Other	no	no
18	*Maoriblatta spp.*	Cockroaches	Other	no	no
19	Coleoptera<br>*Aethina tumida*	Small hive beetle	Other	yes	no
20	*Mecodema spp.*	Ground beetles	Bee associate	no	yes
21	*Scolopterus penicillatus*	Black spined weevil	Other	no	no
22	Dermaptera<br>*Forficula auricularia*	European earwig	Other	yes	no
23	Diptera<br>*Braula coeca*	Bee louse	Bee associate	no	no
24	*Drosophila melanogaster*	Fruit fly	Other	yes	no
25	*Exaireta spinigera*	Garden soldier fly	Other	no	yes
26	*Hermetia illucens*	Black soldier fly	Other	yes	no
27	*Leptotarsus spp.*	Crane fly	Other	no	yes
28	*Sarcophaga crassipalpis*	Flesh fly	Other	no	no
29	Hymenoptera<br>*Apis cerana*	Asiatic honey bee	Bee	yes	no
30	*Apis dorsata*	Giant honey bee	Bee	yes	yes
31	*Apis florea*	Little honey bee	Bee	yes	yes
32	*Apis mellifera*	Honey bee	Bee	yes	yes
33	*Bombus ignitus*	Fiery-tailed bumble bee	Bee	yes	yes
34	*Bombus impatiens*	Common eastern bumble bee	Bee	yes	no
35	*Bombus lapidarius*	Red tailed bumble bee	Bee	yes	yes
36	*Bombus pascuorum*	Common carder bee	Bee	yes	no
37	*Bombus sylvicola*	Forest bumble bee	Bee	yes	yes
38	*Bombus terrestris*	Buff-tailed bumble bee	Bee	yes	no
39	*Linepithema humile*	Argentine ant	Bee associate	yes	yes
40	*Monomorium pharaonis*	Pharaoh ant	Other	yes	yes
41	*Pheidole megacephala*	African big-headed ant	Bee associate	no	yes
42	*Polistes canadensis*	Red paper wasp	Bee associate	yes	no
43	*Polistes chinensis*	Asian paper wasp	Bee associate	no	yes
44	*Polistes fuscatus*	Common paper wasp	Bee associate	yes	no
45	*Polistes humilis*	Australian paper wasp	Bee associate	no	yes
46	*Polistes dominula*	European paper wasp	Bee associate	yes	no
47	*Sphictostethus nitidus*	Golden hunter wasp	Bee associate	no	yes
48	*Solenopsis invicta*	Red fire ant	Bee associate	yes	no

49	*Vespa velutina*	Yellow-legged hornet	Other	yes	yes
50	*Vespula germanica*	German wasp	Bee associate	yes	yes
51	*Vespula vulgaris*	Common wasp	Bee associate	yes	no
52	*Xanthocryptus novozealandicus*	Lemon tree borer parasite	Other	no	yes
53	Lepidoptera<br>*Achroia grisella*	Lesser wax moth	Other	yes	
54	*Danaus plexippus*	Monarch butterfly	Other	yes	yes
55	*Dodonidia helmsii*	Forest ringlet	Conservation concern	no	yes
56	*Galleria mellonella*	Greater wax moth	Bee associate	yes	no
57	*Pieris rapae*	Cabbage white butterfly	Other	yes	yes
58	*Vanessa cardiu*	Painted lady	Other	yes	yes
59	*Vanessa gonerilla*	New Zealand red admiral	Conservation concern	no	yes
60	*Vitula serratilineella*	Dried-fruit moth	Conservation concern	no	no
61	Odonata<br>*Austrolestes colensonis*	Blue damselfly	Conservation concern	no	no
62	Orthoptera<br>*Acheta domestica*	House cricket	Other	yes	no
63	*Bobilla sp.*	Small field cricket	Other	no	no
64	*Deinacrida spp.*	Giant weta	Conservation concern	no	no
65	*Hemideina spp.*	Tree weta	Other	no	no
66	*Hemiandrus spp.*	Ground weta	Other	no	no
67	*Locusta migratoria*	Migratory locust	Other	yes	no
68	*Motuweta spp.*	Tusked weta	Conservation concern	no	no
69	*Pachyrhamma acanthoceras*	Cave weta	Conservation concern	no	no
70	*Teleogryllus commodus*	Black field cricket	Other	no	no
71	*Teleogryllus occipitalis*	Asian cricket	Other	yes	no
72	Phasmatodea<br>*Clitarchus hookeri*	Smooth stick insect	Other	yes	no<br>no
73	Pseudoscorpiones<br>*Chelifer cancroides*	House pseudoscorpion	Other	no	no
74	*Heterochernes novaezealandiae*	Pseudoscorpion	Bee associate	no	no
75	*Nesochernes gracilis*	Pseudoscorpion	Bee associate	no	no
76	Strepsiptera<br>*Stylops melittae*	Twisted-wing parasite	Bee associate	no	no

Supplemental Table S2. BUSCO evaluations of genomic and transcriptomic resources used for the determination of off-target effects of vadescana. C: Complete S: Single copy; D: Duplicated; F: Fragmented; M: Missing; n: Number of genes in OrthoDb lineage.

Class	Order	Species name	Common name	GenBank/RefSeq Genome ID	BUSCO OrthoDB lineage	Estimated BUSCO completeness of genome	Full genome BUSCO scores	Estimated BUSCO completeness of transcriptome	Full transcriptome BUSCO scores
Arachnida	Mesostigmata	*Stratiolaelaps scimitus*	Stratiolaelaps mite	GCA_019614645.1	arachnida_odb10	**98.2%**	C:98.2%[S:97.0%,D:1.2%],F:0.6%,M:1.2%,n:2934		
Arachnida	Mesostigmata	*Tropilaelaps mercedesae*	Tropilaelaps mite	GCA_002081605.1	arachnida_odb10	**97.0%**	C:97.0%[S:95.9%,D:1.1%],F:1.7%,M:1.3%,n:2934	**95.7%**	C:95.7%[S:91.2%,D:4.5%],F:2.2%,M:2.1%,n:2934
Arachnida	Mesostigmata	*Varroa destructor*	Honey bee mite	GCF_002443255.1	arachnida_odb10	**98.9%**	C:98.9%[S:97.8%,D:1.1%],F:0.5%,M:0.6%,n:2934	**99.2%**	C:99.2%[S:38.1%,D:61.1%],F:0.0%,M:0.8%,n:2934
Arachnida	Mesostigmata	*Varroa jacobsoni*	Varroa mite	GCF_002532875.1/GCF_002532875.2	arachnida_odb10	**99.0%**	C:99.0%[S:98.2%,D:0.8%],F:0.4%,M:0.6%,n:2934	**99.6%**	C:99.6%[S:45.9%,D:53.7%],F:0.0%,M:0.4%,n:2934
Arachnida	Trombidiformes	*Acarapis woodi*	Honey bee tracheal mite	GCA_023170135.1	arachnida_odb10	**74.5%**	C:74.5%[S:72.5%,D:2.0%],F:3.9%,M:21.6%,n:2934		
Arachnida	Trombidiformes	*Tetranychus urticae*	Two-spotted spider mite	GCF_000239435.1	arachnida_odb10	**94.6%**	C:94.6%[S:88.9%,D:5.7%],F:0.7%,M:4.7%,n:2934	**94.9%**	C:94.9%[S:71.4%,D:23.5%],F:0.4%,M:4.7%,n:2934
Insecta	Blattodea	*Blattella germanica*	German cockroach	GCA_000762945.2	insecta_odb10	**96.3%**	C:96.3%[S:93.9%,D:2.4%],F:2.8%,M:0.9%,n:1367		
Insecta	Coleoptera	*Aethina tumida*	Small hive beetle	GCF_024364675.1	insecta_odb10	**99.5%**	C:99.5%[S:97.4%,D:2.1%],F:0.	**99.6%**	C:99.6%[S:74.1%,D:25.5%],F:0

							0%,M:0.5%,n:1367		.0%,M:0.4%,n:1367
Insecta	Dermaptera	*Forficula auricularia*	European earwig	GCA_024734495.1	insecta_odb10	**87.2%**	C:87.2%[S:83.0%,D:4.2%],F:3.1%,M:9.7%,n:1367		
Insecta	Diptera	*Drosophila melanogaster*	Fruit fly	GCF_000001215.4	diptera_odb10	**98.7%**	C:98.7%[S:98.5%,D:0.2%],F:0.5%,M:0.8%,n:3285	**99.9%**	C:99.9%[S:40.3%,D:59.6%],F:0.1%,M:0.0%,n:3285
Insecta	Diptera	*Hermetia illucens*	Black soldier fly	GCF_905115235.1	diptera_odb10	**95.3%**	C:95.3%[S:94.6%,D:0.7%],F:1.1%,M:3.6%,n:3285	**98.1%**	C:98.1%[S:63.7%,D:34.4%],F:0.1%,M:1.8%,n:3285
Insecta	Hymenoptera	*Apis cerana*	Asiatic honey bee	GCF_001442555.1	hymenoptera_odb10	**93.6%**	C:93.6%[S:93.2%,D:0.4%],F:2.6%,M:3.8%,n:5991	**94.2%**	C:94.2%[S:51.8%,D:42.4%],F:3.2%,M:2.6%,n:5991
Insecta	Hymenoptera	*Apis dorsata*	Giant honey bee	GCF_000469605.1	hymenoptera_odb10	**96.1%**	C:96.1%[S:96.0%,D:0.1%],F:1.0%,M:2.9%,n:5991	**97.2%**	C:97.2%[S:55.7%,D:41.5%],F:1.0%,M:1.8%,n:5991
Insecta	Hymenoptera	*Apis florea*	Little honey bee	GCF_000184785.3	hymenoptera_odb10	**94.9%**	C:94.9%[S:94.8%,D:0.1%],F:2.3%,M:2.8%,n:5991	**97.0%**	C:97.0%[S:63.4%,D:33.6%],F:1.8%,M:1.2%,n:5991
Insecta	Hymenoptera	*Apis mellifera*	Honey bee	GCF_003254395.2	hymenoptera_odb10	**97.7%**	C:97.7%[S:97.6%,D:0.1%],F:0.3%,M:2.0%,n:5991	**99.0%**	C:99.0%[S:49.3%,D:49.7%],F:0.3%,M:0.7%,n:5991
Insecta	Hymenoptera	*Bombus ignitus*	Fiery-tailed bumble bee	GCA_014825875.1	hymenoptera_odb10	**96.3%**	C:96.3%[S:96.1%,D:0.2%],F:1.2%,M:2.5%,n:5991		
Insecta	Hymenoptera	*Bombus impatiens*	Common eastern bumble bee	GCF_000188095.3	hymenoptera_odb10	**97.4%**	C:97.4%[S:97.3%,D:0.1%],F:0.6%,M:2.0%,n:5991	**99.4%**	C:99.4%[S:46.1%,D:53.3%],F:0.3%,M:0.3%,n:5991
Insecta	Hymenoptera	*Bombus lapidarius*	Red tailed bumble bee	GCA_936014575.1	hymenoptera_odb10	**97.6%**	C:97.6%[S:97.2%,D:0.4%],F:0.4%,M:2.0%,n:5991		
Insecta	Hymenoptera	*Bombus pascuorum*	Common carder bee	GCF_905332965.1	hymenoptera_odb10	**97.8%**	C:97.8%[S:97.5%,D:0.3%],F:0.	**99.5%**	C:99.5%[S:50.7%,D:48.8%],F:0

							4%,M:1.8%,n:5991		.2%,M:0.3%,n:5991
Insecta	Hymenoptera	*Bombus sylvicola*	Forest bumble bee	GCA_019677175.1	hymenoptera_odb10	**97.1%**	C:97.1%[S:96.9%,D:0.2%],F:0.4%,M:2.5%,n:5991		
Insecta	Hymenoptera	*Bombus terrestris*	Buff-tailed bumble bee	GCF_910591885.1	hymenoptera_odb10	**97.6%**	C:97.6%[S:97.3%,D:0.3%],F:0.5%,M:1.9%,n:5991	**99.3%**	C:99.3%[S:44.0%,D:55.3%],F:0.1%,M:0.6%,n:5991
Insecta	Hymenoptera	*Linepithema humile*	Argentine ant	GCF_000217595.1	hymenoptera_odb10	**96.5%**	C:96.5%[S:96.2%,D:0.3%],F:0.8%,M:2.7%,n:5991	**99.1%**	C:99.1%[S:57.2%,D:41.9%],F:0.4%,M:0.5%,n:5991
Insecta	Hymenoptera	*Monomorium pharaonis*	Pharaoh ant	GCF_013373865.1	hymenoptera_odb10	**96.9%**	C:96.9%[S:94.4%,D:2.5%],F:0.7%,M:2.4%,n:5991	**99.4%**	C:99.4%[S:48.1%,D:51.3%],F:0.3%,M:0.3%,n:5991
Insecta	Hymenoptera	*Polistes canadensis*	Red paper wasp	GCF_001313835.1	hymenoptera_odb10	**95.5%**	C:95.5%[S:95.1%,D:0.4%],F:1.3%,M:3.2%,n:5991	**98.0%**	C:98.0%[S:55.1%,D:42.9%],F:0.7%,M:1.3%,n:5991
Insecta	Hymenoptera	*Polistes dominula*	European paper wasp	GCF_001465965.1	hymenoptera_odb10	**95.8%**	C:95.8%[S:95.5%,D:0.3%],F:1.0%,M:3.2%,n:5991	**98.2%**	C:98.2%[S:53.4%,D:44.8%],F:0.4%,M:1.4%,n:5991
Insecta	Hymenoptera	*Polistes fuscatus*	Common paper wasp	GCF_010416935.1	hymenoptera_odb10	**95.6%**	C:95.6%[S:95.2%,D:0.4%],F:1.3%,M:3.1%,n:5991	**98.0%**	C:98.0%[S:54.2%,D:43.8%],F:0.4%,M:1.6%,n:5991
Insecta	Hymenoptera	*Solenopsis invicta*	Red fire ant	GCF_016802725.1	hymenoptera_odb10	**96.7%**	C:96.7%[S:96.1%,D:0.6%],F:0.8%,M:2.5%,n:5991	**99.6%**	C:99.6%[S:47.6%,D:52.0%],F:0.3%,M:0.1%,n:5991
Insecta	Hymenoptera	*Vespa velutina*	Yellow-legged hornet	GCF_912470025.1	hymenoptera_odb10	**96.4%**	C:96.4%[S:96.2%,D:0.2%],F:0.9%,M:2.7%,n:5991	**97.8%**	C:97.8%[S:37.1%,D:60.7%],F:0.6%,M:1.6%,n:5991
Insecta	Hymenoptera	*Vespula germanica*	German wasp	GCA_905340365.1	hymenoptera_odb10	**96.4%**	C:96.4%[S:96.2%,D:0.2%],F:0.9%,M:2.7%,n:5991		
Insecta	Hymenoptera	*Vespula vulgaris*	Common wasp	GCF_905475345.1	hymenoptera_odb10	**96.5%**	C:96.5%[S:96.4%,D:0.1%],F:0.	**98.5%**	C:98.5%[S:44.2%,D:54.3%],F:0

							8%,M:2.7%,n:5991		.2%,M:1.3%,n:5991
Insecta	Lepidoptera	*Achroia grisella*	Lesser wax moth	GCF_030625045.1	lepidoptera_odb10	**98.7%**	C:98.7%[S:96.1%,D:2.6%],F:0.3%,M:1.0%,n:5286	**99.4%**	C:99.4%[S:77.2%,D:22.2%],F:0.1%,M:0.5%,n:5286
Insecta	Lepidoptera	*Danaus plexippus*	Monarch butterfly	GCF_009731565.1	lepidoptera_odb10	**98.3%**	C:98.3%[S:97.9%,D:0.4%],F:0.7%,M:1.0%,n:5286	**98.9%**	C:98.9%[S:71.5%,D:27.4%],F:0.5%,M:0.6%,n:5286
Insecta	Lepidoptera	*Galleria mellonella*	Greater wax moth	GCF_026898425.1	lepidoptera_odb10	**98.5%**	C:98.5%[S:97.5%,D:1.0%],F:0.4%,M:1.1%,n:5286	**99.4%**	C:99.4%[S:65.3%,D:34.1%],F:0.1%,M:0.5%,n:5286
Insecta	Lepidoptera	*Pieris rapae*	Cabbage white butterfly	GCF_905147795.1	lepidoptera_odb10	**98.8%**	C:98.8%[S:98.4%,D:0.4%],F:0.2%,M:1.0%,n:5286	**98.6%**	C:98.6%[S:67.6%,D:31.0%],F:0.2%,M:1.2%,n:5286
Insecta	Lepidoptera	*Vanessa cardiu*	Painted lady	GCF_905220365.1	lepidoptera_odb10	**98.7%**	C:98.7%[S:98.6%,D:0.1%],F:0.3%,M:1.0%,n:5286	**99.4%**	C:99.4%[S:73.7%,D:25.7%],F:0.1%,M:0.5%,n:5286
Insecta	Orthoptera	*Acheta domestica*	House cricket	GCA_014858955.1	insecta_odb10	**48.7%**	C:48.7%[S:47.5%,D:1.2%],F:35.6%,M:15.7%,n:1367		
Insecta	Orthoptera	*Locusta migratoria*	Migratory locust	GCA_026315105.1	insecta_odb10	**86.1%**	C:86.1%[S:82.3.5%,D:3.8%],F:6.7%,M:7.2%,n:1367		
Insecta	Orthoptera	*Teleogryllus occipitalis*	Asian cricket	GCA_011170035.1	insecta_odb10	**95.9%**	C:95.9%[S:93.3%,D:2.6%],F:2.1%,M:2.0%,n:1367		
Insecta	Phasmatodea	*Clitarchus hookeri*	Smooth stick insect	GCA_002778355.1	insecta_odb10	**96.2%**	C:96.2%[S:92.2%,D:4.0%],F:2.7%,M:1.1%,n:1367		
